# The effect of an online acceptance and commitment intervention on the meaning-making process in cancer patients following hematopoietic cell transplantation: study protocol for a randomized controlled trial enhanced with single-case experimental design

**DOI:** 10.1186/s13063-024-08235-1

**Published:** 2024-06-18

**Authors:** Aleksandra Kroemeke, Joanna Dudek, Marta Kijowska, Ray Owen, Małgorzata Sobczyk-Kruszelnicka

**Affiliations:** 1 Institute of Psychology, Health & Coping Research Group, SWPS University, Warsaw, Poland; 2 Faculty of Psychology, SWPS University, Warsaw, Poland; 3 Gloucester, UK; 4 Department of Bone Marrow Transplantation and Oncohematology, Maria Sklodowska-Curie National Research Institute of Oncology Gliwice Branch, Gliwice, Poland

**Keywords:** Meaning-making, Meanings made, Well-being, Acceptance and Commitment Therapy, Cancer, Hematopoietic cell transplantation, Randomized controlled trial, Single-case experimental design

## Abstract

**Background:**

Hematopoietic cell transplantation (HCT) is a highly invasive and life-threatening treatment for hematological neoplasms and some types of cancer that can challenge the patient’s meaning structures. Restoring meaning (i.e., building more flexible and significant explanations of the disease and treatment burden) can be aided by strengthening psychological flexibility by means of an Acceptance and Commitment Therapy (ACT) intervention. Thus, this trial aims to examine the effect of the ACT intervention on the meaning-making process and the underlying mechanisms of change in patients following HCT compared to a minimally enhanced usual care (mEUC) control group. The trial will be enhanced with a single-case experimental design (SCED), where ACT interventions will be compared between individuals with various pre-intervention intervals.

**Methods:**

In total, 192 patients who qualify for the first autologous or allogeneic HCT will be recruited for a two-armed parallel randomized controlled trial comparing an online self-help 14-day ACT training to education sessions (recommendations following HCT). In both conditions, participants will receive once a day a short survey and intervention proposal (about 5–10 min a day) in the outpatient period. Double-blinded assessment will be conducted at baseline, during the intervention, immediately, 1 month, and 3 months after the intervention. In addition, 6–9 participants will be invited to SCED and randomly assigned to pre-intervention measurement length (1–3 weeks) before completing ACT intervention, followed by 7-day observations at the 2nd and 3rd post-intervention measure. The primary outcome is meaning-related distress. Secondary outcomes include psychological flexibility, meaning-making coping, meanings made, and well-being as well as global and situational meaning.

**Discussion:**

This trial represents the first study that integrates the ACT and meaning-making frameworks to reduce meaning-related distress, stimulate the meaning-making process, and enhance the well-being of HCT recipients. Testing of an intervention to address existential concerns unique to patients undergoing HCT will be reinforced by a statistically rigorous idiographic approach to see what works for whom and when. Since access to interventions in the HCT population is limited, the web-based ACT self-help program could potentially fill this gap.

**Trial registration:**

ClinicalTrials.gov ID: NCT06266182. Registered on February 20, 2024.

## Administrative information

Note: the numbers in curly brackets in this protocol refer to SPIRIT checklist item numbers. The order of the items has been modified to group similar items (see http://www.equator-network.org/reporting-guidelines/spirit-2013-statement-defining-standard-protocol-items-for-clinical-trials/).

**Table:** 

Title {1}	The Effect of an online Acceptance and Commitment Intervention on the Meaning-Making Process in Cancer Patients following Hematopoietic Cell Transplantation: Study Protocol for a Randomized Controlled Trial enhanced with Single-case Experimental Design
Trial registration {2a and 2b}.	ClinicalTrials.gov ID: NCT06266182
Protocol version {3}	Version 3.0 dated May 13, 2024.
Funding {4}	The work is supported by the National Science Centre, Poland [grant number 2020/39/B/HS6/01927 awarded to AK].
Author details {5a}	^1^SWPS University, Institute of Psychology, Health & Coping Research Group, Poland; ^2^SWPS University, Faculty of Psychology in Warsaw, Poland; ^3^Private Practice, UK; ^4^Maria Sklodowska-Curie National Research Institute of Oncology Gliwice Branch, Department of Bone Marrow Transplantation and Oncohematology, Poland
Name and contact information for the trial sponsor {5b}	National Science Centre, Poland; biuro@ncn.gov.pl
Role of sponsor {5c}	The funders had no role in study design, data collection, analysis, and interpretation, decision to publish, or preparation of the manuscript

## Introduction

### Background and rationale {6a}

Hematologic neoplasms (e.g., lymphomas or acute leukemias) due to unique and sometimes increased challenges are highly stressful conditions. Treatment-related challenges can impede the realization of life goals and violate general beliefs and a sense of meaning as defined by the integrative meaning-making model [1]. A significant point on the trajectory of coping, challenging the patient meaning structures, may be hematopoietic cell transplantation (HCT). HCT is a highly invasive and life-threatening treatment for hematological neoplasms and some types of cancer (e.g., testicular cancer). In the acute phase, HCT involves the destruction of the patient hematopoietic system through radio and/or chemotherapy and then its restoration via autologous or allogeneic cell transplantation [2, 3]. During in- and outpatient conditions, patients usually experience burdensome adverse effects and have to follow strong medical regimens [2, 4]. Evidence suggests that HCT affects a patient physical (e.g., fatigue), psychological (e.g., anxiety and depression symptoms), social (e.g., financial concerns, employment disruptions), and spiritual (e.g., existential concerns) well-being [5]. HCT recipients may confront fear of death, loss of control, feelings of uncertainty and social isolation, increased dependence, or disabling physical symptoms in the short and long term after transplantation [6, 7]. Some models of adaptation and adjustment argue that restoring meaning is central to adapting to these conditions [8].

### Meaning-making process following HCT

The most commonly mentioned factors of meaning reconstruction are meaning-making coping and meanings made [1, 9]. Meaning-making is related to the process of searching for meaning and explanation for adversity (i.e., seeking understanding of disease), whereas meanings made is the product of the meaning-making process (i.e., giving meaning to the disease, acceptance, finding benefits, or change of identity due to disease). According to the integrative meaning-making model [1, 10], distress related to the discrepancy between global meaning (i.e., basic goals and beliefs, and fundamental assumptions about life) and situational meaning (i.e., the personal significance of a particular situation) initiates meaning-making coping, which impacts the meanings made and then well-being. However, a prolonged unsuccessful search for meaning can be maladaptive. Indeed, the adaptability of meaning-making coping depends on whether the meaning has been found or restored [10].

A review of the narratives shows that HCT recipients who were able to find meaning in their experience were better able to cope with physical symptoms and were less likely to report unfavorable psychological outcomes after transplant than those who had difficulty finding meaning [6]. Meanings made was also an essential link connecting meaning-making and well-being in HCT recipients in a daily diary study lasting 28 days after hospital discharge [11]. The direct effect of average meaning-making coping was unfavorable but positive when mediated by meanings made. In another study among HCT recipients in the late outpatient period with a 4-month follow-up interval, only changes in meaning-making coping were associated with changes in well-being, and these correlates were positive and negative [12]. The role of meanings made in these relationships was, however, not tested. Indeed, few studies tested the assumptions of the integrative meaning-making model in the context of HCT. More often, the focus is on the global meaning which turns out to be a dynamic construct. In a longitudinal study, sense of meaning decreased 1 month post-HCT and returned to pre-transplant levels by 6 months post-HCT. Moreover, a greater pre-HCT sense of meaning predicted more favorable psychological and physical outcomes during the 12 months following HCT [13].

Hence, an intervention targeting the ability to successfully search for meaning and find it holds promise in terms of facilitating recovery following HCT and adjustment. To date, no trials tested such interventions among patients undergoing HCT. To the best of our knowledge, two studies are currently underway in HCT recipients that include modules directed at searching for meaning i.e., identifying benefits and meaning. The first one examines the effect of one-on-one, in-person intervention promoting resilience in stress management [14], whereas the second is a phone-delivered positive psychology intervention [15]. Both, however, will not evaluate the outcomes from the perspective of the meaning-making model. A systematic review shows that various psychosocial interventions can promote meaning and purpose in the cancer population [16]. Nevertheless, these targeting meaning enhancements demonstrate a higher effect size. One of the promising approaches potentially fostering meaning-making in disease is Acceptance and Commitment Therapy [17].

### Acceptance and Commitment Therapy (ACT) intervention

Acceptance and Commitment Therapy (ACT) is a transdiagnostic therapeutic approach rooted in the contextual behavioral science that aims to improve the psychological functioning and well-being of individual by increasing psychological flexibility (i.e., the ability to engage in values-based actions even in the presence of unpleasant or difficult experiences) [17]. To achieve this goal ACT targets six core processes: (*1*) contact with the present moment—paying attention to different aspects of the internal and external environment; (*2*) self-as-context—the ability to look at one’s internal experiences from a broader perspective; (*3*) acceptance—making room for thoughts, feelings, and sensations, even those that are unpleasant; (*4*) defusion—noticing thoughts instead of being controlled by them; (*5*) values—knowing what really matters; and (*6*) committed action—taking values-congruent actions even in the presence of difficulties. During the therapy, the individual learns to assess the workability of strategies used to cope with difficult, unwanted private experiences and to use mindfulness and acceptance skills when necessary. Those skills allow the individual to recognize moments when they have an opportunity to engage in behaviors consistent with their values and fully immerse themselves in those activities, even in the presence of painful thoughts, feelings, or sensations. Individuals are not asked to accept painful private experiences (e.g., physical pain) if there is an effective way to get rid of the pain; acceptance means embracing painful private experiences only when there is no effective way of escaping painful experiences on a long-term basis or when the means of escape comes at too high a cost in terms of valued living. Techniques used in ACT to obtain the aforementioned changes include using metaphors, experiential exercises, and functional analysis [17].

Besides the typical use of ACT as an individual face-to-face therapy, ACT was also tested in a group format (e.g., for anxiety and depression [18] or chronic pain [19]), as a self-help form [20] as well as technology-supported intervention (using online materials, web or phone applications, telephone) with or without therapeutic guidance [21].

ACT has been proven to be an effective intervention for various conditions [22], with the growing number of randomized controlled trials [23] and mediational studies showing that psychological flexibility is a mediator of the intervention [24]. Several systematic reviews and metanalyses provide evidence for ACT effectiveness in improving the quality of life and decreasing psychological distress among cancer patients [25–29]. Other systematic reviews support ACT efficacy in improving quality of life and symptoms for long-term chronic conditions [30, 31], also including the technology-supported delivery of ACT [21]. Finally, ACT is considered to be an effective treatment for chronic pain, being recognized by the American Psychological Association as an evidence-based treatment with “strong research support” [32].

### The links between ACT and the meaning-making process

ACT and meaning-making frameworks share common philosophical roots, including constructivism and existentialism [9]. The ACT model promotes acceptance of what is difficult to change or is not subject to change (such as chronic disease or burden of toxic treatment), taking responsibility for one’s own experiences and actions and creating a meaningful life by engaging in activities that match one’s values [33]. While meaning-making is not an explicit goal of ACT, creating psychological flexibility should foster meaning-making in disease or following HCT by building more flexible and workable meaning-making explanations of disease [34]. ACT emphasizes increased awareness of what matters most to the individual and a stepping back from automatic patterns of thought and behavior. Both of these abilities should facilitate meaning-making, i.e., changing global meaning or a reappraisal of situational meaning to achieve congruence, thus alleviating the distress of the event such as HCT. Achieving congruence should end meaning-making coping and be associated with meanings made and improved well-being.

### Objectives {7}

This trial aims to examine the effect of an online self-help ACT intervention on the meaning-making process and the underlying mechanisms of change in patients following HCT compared to a minimally enhanced usual care (mEUC) control group. The trial will be enhanced with a single-case experimental design (SCED), where ACT interventions will be compared between individuals with various pre-intervention intervals. As the change process is characterized by complexity, traditional examination of intervention efficacy will be enriched with a temporal perspective (i.e., examination of trajectories of change in primary and secondary outcomes over time) and a systems perspective (i.e., network analysis depicting the pattern of connections between components of the system). The latter assumes that an intervention transforms the connectivity of the networks of intervention goals, the outcome of the intervention, and the connections between the two networks [35, 36].

It is hypothesized that the ACT intervention group would show increased psychological flexibility and decreased meaning-related distress compared with the control group (hypothesis 1). Additionally, an increase in meanings made and well-being is anticipated (hypothesis 2). In more exploratory terms, the moderating effect of individual resources (i.e., global and situational meaning, baseline well-being) and demographic and clinical factors on the effect of the intervention will also be examined. Moreover, it is hypothesized that psychological flexibility and meaning-making coping would mediate the ACT intervention effects on meaning-related distress, meanings made, and well-being in HCT recipients (hypothesis 3). Finally, following the network theory, it is hypothesized that the ACT intervention group will display more robust positive connections within the psychological flexibility and meaning-making coping network (hypothesis 4), weaker connections within the distress network (hypothesis 5), more negative connections of distress with psychological flexibility and meaning-making coping (hypothesis 6), and more positive connections between psychological flexibility, meaning-making coping, meanings made, and well-being as compared to control conditions (hypothesis 7).

### Trial design {8}

A two-armed parallel randomized controlled trial (RCT) will be conducted to determine the effects of an online Acceptance and Commitment Therapy ACT intervention on the meaning-making process in patients following HCT. Participants will be randomly assigned in a double-blinded manner to ACT intervention and education conditions at a ratio of 1:1. RCT will be enhanced with a randomized multiple-baseline single-case experimental design (SCED). SCED will proceed according to the AB + post-intervention design, where A is the pre-intervention phase and B is the intervention phase, followed by the post-intervention phase. Participants will be randomly assigned to one of three pre-intervention measurement lengths (7 days, 14 days, 21 days) followed by 7-day observations at the 2nd and 3rd post-intervention measure.

## Methods: participants, interventions and outcomes

### Study setting {9}

Recruitment will take place in the Department of Bone Marrow Transplantation and Oncohematology of the Maria Sklodowska-Curie National Research Institute of Oncology (MSCNRIO) Gliwice Branch. MSCNRIO branch in Gliwice is the leading facility in Poland that performs HCT. Approximately 150 primary transplants are performed there annually (approx. 200 HCT in total).

### Eligibility criteria {10}

The participation criteria will include (*a*) qualification for the first autologous or allogeneic HCT due to hematologic malignancies or solid tumors, (*b*) age ≥ 18 years, (*c*) signed written informed consent, (*d*) ability to read and write in Polish, and (*e*) daily access to the Internet by computer and/or mobile device. The exclusion criteria will be as follows: (*a*) major psychiatric or cognitive disorder that would impede providing informed consent and study participation, (*b*) inability to cooperate and give informed consent, (*c*) hearing, seeing, or movement impairment that precludes participation, (*d*) current participation in any form of psychotherapy, (*e*) no access to the Internet and computer and/or mobile device, and (*f*) inability to use a computer and/or mobile device and the Internet.

### Who will take informed consent? {26a}

Written informed consent to participate in the study will be obtained by the recruiter (member of the research team), in direct contact with the participant and after an extensive briefing.

### Additional consent provisions for collection and use of participant data and biological specimens {26b}

N/A. Biological specimens will not be collected.

## Interventions

### Explanation for the choice of comparators {6b}

In RCT, the ACT intervention will be compared with minimally enhanced usual care (mEUC). Standard psychological care following HCT does not include a standard psychological care protocol. Psychological care for HCT recipients is provided if needed according to the physician’s recommendation in the event of the patient’s functioning deteriorating. Thus, to maintain the same conditions in both trials, participants in the control condition will receive cognitively neutral tasks (education) from which no effects are expected for the meaning-making process. In SCED, comparisons between participants with different pre-intervention measurement lengths will be conducted.

### Intervention description {11a}

*ACT intervention* “The Path to Health” will start on the second day after hospital discharge for individuals in RCT or after 7–21-day pre-intervention measurement in individuals in SCED. It will take 14 days (+ day 0 with organizational information). Each day, participants will receive a web-based intervention consisting of the theoretical introduction (including examples of patients’ experiences and metaphors) and practical ACT activity (e.g., reflective questions, experiential exercise, values card sorting test). Most of the activities are followed by a debrief that includes the patient’s reactions to this particular exercise and practical tips. On some days, participants will also receive additional exercise (optional).

Using the metaphor of life as a journey, participants will learn to recognize where they are headed (values), when there is a moment of choice between actions that lead towards values or away from them, and how to use attention flexibly to free themselves from the power of thoughts, to open up and accept emotions so that they can effectively take action in line with their values (Table 1). Each introduction and each activity will be available in written form and audio. The ACT intervention is built from standard ACT activities [37–40] and tailored to the context of the disease and treatment. Participants will be advised to do one activity a day, but they will be able to come back to the chosen activities or practice them a couple of times if necessary.

**Table 1:** Overview of the ACT intervention “*The Path to Health*”

Day	Content	ACT processes	Exercise
0. Practical information	Introduction. General information about psychological flexibility training. Contact information. Resources (helpline). Reflective questions to increase the intrinsic motivation of participants		
1. Where are you going?	“Life as a journey” metaphor: values as the direction, and openness and awareness as skills necessary to move towards chosen direction. Discussing basic “roadblocks” using patient’s examples Initial values assessment	An overview of psychological flexibility, values	Initial values clarification: open-ended/reflective questions
2. Where do roadblocks come from?	Overview of two factors that contribute to mental suffering: the way the human mind functions (Metaphor “Mind as overly helpful passenger”) and the illusion of control. (Un)workability of different strategies of coping with unwanted private experiences	Creative hopelessness, introduction to defusion and acceptance	Checking the workability of personal strategies used to cope with unwanted internal experiences (open-ended questions
3. Notice the Choice Point	Presenting the Choice point, differentiating towards and away moves	Overview of ACT model, committed actions	Creating personal Choice Point
4. Where are you now?	Dropping Anchor—overview and practice (original metaphor was adjusted to the specificity of participants—instead of dropping an anchor in the ocean participants were asked to “pull over” and “take a break in their journey”)	Contact with present moment, self-as-context, acceptance, defusion	Dropping Anchor- modified versions: 3 min and 1 min (participants choose one)
5. Show yourself kindness	Self-compassion—introducing the concept and its purpose, discussing common misunderstandings about self-compassion	Contact with the present moment, self-as-context, acceptance, defusion	Kind hand
6. Focus on the present moment	Flexible attention—definition, purpose, discussing common misunderstandings	Contact with the present moment	Mindful walk, savoring (participants choose one exercise)
7. Look at the bigger picture	Introducing the topic of looking at own internal experiences from a perspective of observing self. Metaphor “The sky and the weather”	Self-as-context	Observing self
8. Notice your thoughts	Presenting the most common difficulties caused by mental activity and related to fusion with thoughts. “Passengers on the bus” metaphor. Introducing defusion	Defusion	“I have a thought…”
9. Look at your thoughts	Deepening the topic of defusion, questions to check thoughts’ workability	Defusion	Leaves on the stream Additional/optional activities: repetition, singing, silly voices, thoughts on a computer screen, thoughts as passing objects, thanking the Mind
10. Acquaint your emotions	Discussing acceptance—the ability to acknowledge, allow, and accommodate internal experiences. “Two sides of the coin” metaphor	Acceptance	Acceptance of emotions
11. Embrace your feelings	Deepening the topic of acceptance. Modified “unwanted guest” metaphor (the metaphor was adjusted to Polish culture). Presenting a list of acceptance metaphors	Acceptance	Choosing a favorite metaphor, consolidating information about acceptance. Additional/optional activities: questions that facilitate acceptance
12. Check what is important for you	Discussing values—what are values how they are different from goals, and how one can choose them	Values	Values checklist Additional/optional activities: questions about values
13. Take a step in the chosen direction	Introduction to committed actions. Planning specific actions consistent with values	Committed actions	Choosing an action that will be consistent with one’s values Additional: Discussing potential barriers and how to deal with them
14. This is just the beginning of the journey	Summary, presenting different options for using presented activities in the future	Psychological flexibility (all ACT processes)	Planning next steps, and conclusions

During the same period, participants allocated to the *education* in RCT will receive an online guide to post-HCT recommendations. Each day, participants will receive information about post-transplant prescriptions along with exercises. Participants will receive guidelines in several areas: diet, physical activity, hygiene, rest, social interactions, and sexual health. During the first 3 days, nutrition will be discussed, including the principles of healthy diet after HCT. On the fourth day, participants will learn the rules of personal hygiene. The fifth day is devoted to presenting the rules aimed at preventing infection. On the sixth day, the issue of body fatigue will be discussed. For the next 3 days, the main topic will be the resumption of activity, mostly physical activity. The tenth day is devoted to safe social contacts. On the eleventh day, participants will work on their sleep. On the twelfth day, sexual health will be discussed. Day 13 is devoted to discussing the issue of rest. And the last day will be a summary of all the guidelines. The exercises serve as an extension of the topic (e.g., watching a video presenting the principles of nutrition) or the emphasis is on practice to support the implementation (e.g., preparing a sequence of exercises and performing them several times a day). The content is prepared based on available guides for HCT recipients. It was also verified by a hemato-oncologist.

### Criteria for discontinuing or modifying allocated interventions {11b}

Modification of assigned interventions is not provided for. Disease recurrence will be the criteria for discontinuation of the intervention. The participant can also discontinue the intervention at any time without any negative consequences.

### Strategies to improve adherence to interventions {11c}

To improve adherence to the intervention, participants will receive daily reminders about the intervention. Also, direct technical support will be available 24/7. If participants drop out or stop using the intervention, they will be asked for the reason(s) why they decided to quit the intervention and/or study.

### Relevant concomitant care permitted or prohibited during the trial {11d}

Individuals participating in any form of psychotherapy will not be eligible for the study. Participation in forms of psychological support will be monitored on an ongoing basis.

### Provisions for post-trial care {30}

Upon completion of the study, all participants will have access to the self-help ACT intervention booklet with written and recorded exercises.

### Outcomes {12}

The primary and secondary outcomes will be assessed at baseline (before HCT), during the intervention, immediately, 1 month, and 3 months after the intervention (Table 2). In SCED, 1 month and 3 months post-intervention assessments will be preceded by 7-day daily diaries. A summary of the outcome measures that will be used in this study is available in Table 3.

**Table 2:**
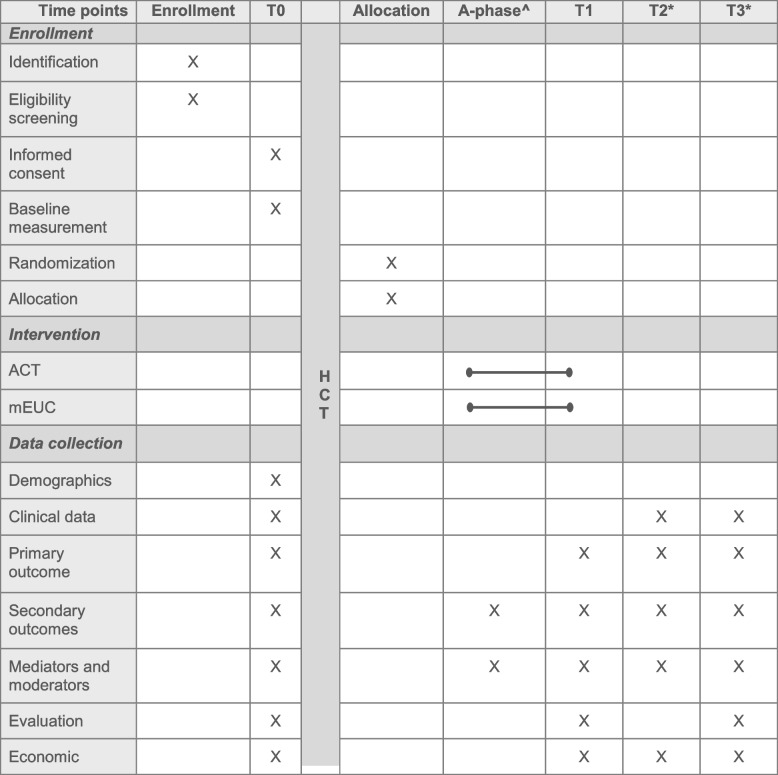
Assessment schedule

*HCT* Hematopoietic cell transplantation, *ACT* Acceptance and commitment therapy intervention, *mEUC* Minimally enhanced usual care control condition (education; in RCT only), *T0* Baseline (hospitalization), *A-phase* Pre-intervention phase in SCED lasting 7, 14, or 21 days, *T1* Post-intervention assessment, *T2* 1-month post-intervention, *T3* 3-month post-intervention

^SCED only

*In SCED, each measurement is preceded by a 7-day diary measurement

**Table 3:** A summary of the outcome measures

Outcome	Component of enhanced meaning-making model	Tool	Variable	Subscales	No. of items	Scale
Primary	Distress	GMVS	Global meaning violation	Belief violation, Goal violation	12	5-point scale
Secondary	Global meaning	B-IPQ	Cognitive and emotional representations of illness	Cognitive illness representation, Emotional illness representation, Illness comprehensibility representation	8	11-point scale
		MLQ	Meaning in life	Presence of meaning	5	7-point scale
	Situational meaning	CSE	Coping self-efficacy	Problem-focused Coping, Emotion-focused Coping, Social Support Coping	6	5-point scale
	Meanings made	C-PTGI-SF	Post-traumatic growth	Relating to Others, New Possibilities, Personal Strength, Spiritual Change, Appreciation of Life	10	6-point scale
		MLC	Meanings made	Sense made, Benefit finding, Sense of identity change	3	5-point scale
	Well-being	PHQ-4	Symptoms of anxiety and depression	Symptoms of anxiety, Symptoms of depression	4	4-point scale
		R-UCLA 3-item Loneliness Scale + R-UCLA 2 items	Perceived social isolation	Social aspect of loneliness (R-UCLA 3-items), Emotional aspect of loneliness (additional 2 items form R-UCLA)	5	4-point scale
		Community Life Survey	Perceived loneliness	n/a	1	4-point scale
		EORTC QLQ-C30	Health-related quality of life	Physical, role, social, emotional, and cognitive functioning, as well as various symptoms, financial impact and global quality of life	30	4-point scale 7-point scale
Mediators/moderators	Psychological flexibility	CompACT-9	Psychological flexibility	Openness to Experience, Behavioral Awareness, Valued Action	9	7-point scale
	Daily psychological flexibility	CompACT-4	Daily psychological flexibility	Daily openness to Experience, Daily behavioral Awareness, Daily valued Action	4	7-point scale
	Meaning-making	CBI	Deliberate meaning-making coping	n/a	9	6-point scale
		ERRI: Intrusive ruminations subscale	Automatic meaning-making coping	n/a	10	4-point scale
		MLQ	Meaning in life	Searching for meaning	5	7-point scale
	Daily meaning-making	ERRI	Daily meaning-making coping	Daily deliberate meaning-making coping, Daily automatic meaning-making coping	4	4-point scale
	Daily meanings made	MLC	Daily meanings made	Daily sense made, Daily benefit finding, Daily sense of identity change	3	5-point scale
	Daily subjective health		Daily subjective health	n/a	1	5-point scale
	Daily affect		Daily affect	Daily positive affect, Daily negative affect	4	5-point scale

*B-IPQ* Brief-Illness Perception Questionnaire, *CBI* Core Beliefs Inventory, *CompACT-9* Comprehensive Assessment of Acceptance and Commitment Therapy Processes, *C-PTGI-SF* the “current standing” Post-Traumatic Growth Inventory-Short Form, *CSE* Coping Self-Efficacy Scale, *EORTC QLQ-C30* Quality of Life Questionnaire of the European Organization for Research and Treatment of Cancer, *ERRI* Event-Related Rumination Inventory, *GMVS* Global Meaning Violation Scale, *MLC* Meaning of Loss Codebook, *MLQ* Meaning in Life Questionnaire, *PHQ-4* Patient Health Questionnaire, *R-UCLA* Revised UCLA Loneliness Scale

#### Primary outcomes

The primary outcome will be the changes compared to the baseline in meaning-related distress as assessed by the Global Meaning Violation Scale (GMVS) [41].

#### Secondary outcomes

The secondary outcomes will be changes from baseline in global meaning, situational meaning, meanings made, and well-being. Global meaning will be measured by cognitive and emotional representations of illness and global presence of meaning using the Brief-Illness Perception Questionnaire (B-IBP) [42] and Meaning in Life Questionnaire (MLQ) [43], respectively. Coping self-efficacy, an indicator of situational meaning, will be assessed with the Perceived Coping Self-Efficacy (CSE) Scale [44]. Meanings made will be assessed using the “current standing” Post-Traumatic Growth Inventory-Short Form (C-PTGI-SF) [45, 46] and 3-item scale based on the Meaning of Loss Codebook (MLC) [47]. Depressive and anxiety symptoms will be assessed with the Patient Health Questionnaire (PHQ-4) [48], while loneliness, as recommended by the British Office for National Statistics [49], will be evaluated with the enhanced R-UCLA 3-item Loneliness Scale [50] and direct question from the Community Life Survey [51].

#### Mediators and moderators

To assess putative mechanisms of change and change moderators, meaning-making coping and psychological flexibility will be measured longitudinally. In this scheme, deliberate and automatic meaning-making coping will be assessed with the Core Beliefs Inventory (CBI) [52] and the intrusive ruminations subscale from the Event-Related Rumination Inventory (ERRI) [53], respectively. Psychological flexibility will be measured using the Comprehensive Assessment of Acceptance and Commitment Therapy Processes (CompACT-9) [54]. In addition, fluctuations in meaning-making coping, meanings made, psychological flexibility, and well-being (i.e., subjective health and positive and negative affect) will be measured in an intensive longitudinal manner (i.e., daily) throughout the intervention in RCT and pre- to post-intervention in SCED. Daily meaning-making coping (deliberate and automatic) will be measured with an abbreviated and tailored to the daily measurement and context of the study 4-item version of the ERRI questionnaire. Daily meanings made will be evaluated using a contextualized 3-item scale based on the Meaning of Loss Codebook (MLC). Daily psychological flexibility will be measured using a shortened to 4-item version of the CompACT questionnaire. Daily subjective health will be assessed by a single-item statement “Generally, I can say my health today was…” on a 5-point scale ranging from 1 (bad) to 5 (excellent). Daily positive and negative affect will be assessed with two positive (happy, cheerful) and two negative adjectives (sad, gloomy) based on the Circumplex Model of Emotion [55].

#### Evaluation

Feasibility will be examined via attrition and adherence rates as well as questions about intervention engagement. Acceptability will be measured by intervention satisfaction and evaluation (attractiveness and easiness). Adherence to the intervention will be estimated based on the dropout rate (i.e., the percentage of participants who do not log in to the intervention on a given day) and self-reported questions about engagement in the intervention: (*1*) the number of days on which the proposed exercises were done seriously, (*2*) the number of minutes spent on average in training, and (*3*) the use of various training components. Satisfaction with the intervention will be measured using 4 questions (no. 3, 4, 7, and 8) from the Client Satisfaction Questionnaire (CSQ-8) [56] modified to the intervention context and online form. Evaluation of the intervention will be assessed using questions of the author’s own measuring the ease and attractiveness of the training.

#### Economic

The cost-effectiveness of the intervention will be examined by estimating health-related quality of life as measured by the Quality of Life Questionnaire of the European Organization for Research and Treatment of Cancer (EORTC QLQ-C30) [57].

#### Other measures

At the baseline, demographic data (e.g., age, sex, education, marital status, employment) will also be collected and partially measured using the Diversity Minimal Item Set (DiMIS) [58]. Clinical data (e.g., diagnosis, time since diagnosis, conditioning, concomitant diseases) will be obtained from the medical records.

### Participant timeline {13}

**Fig. 1:**
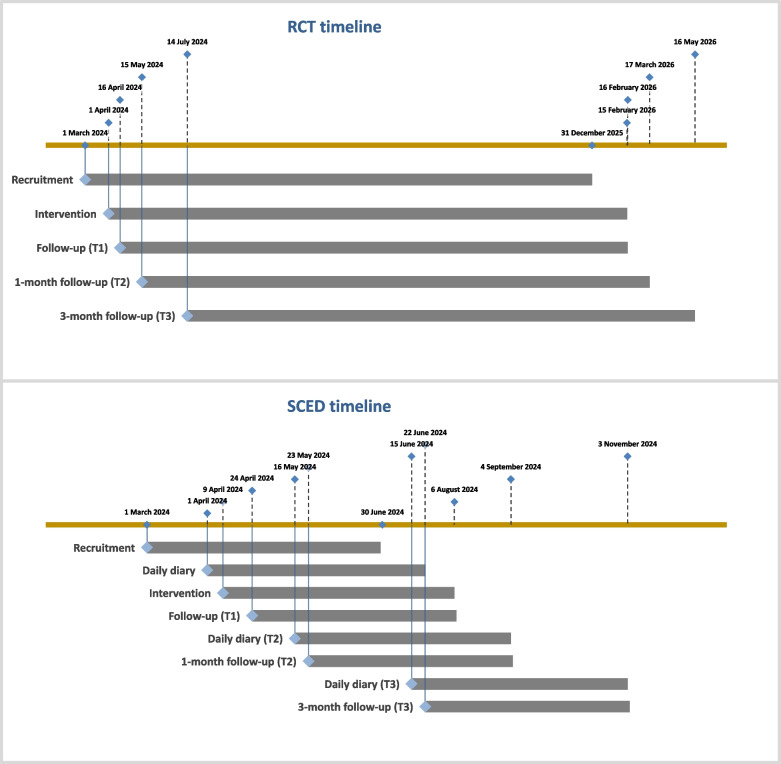
Timeline for RCT and SCED study

Figure 1 describes the project timeline.

### Sample size {14}

In RCT, the sample size was calculated based on an analysis of variance with two groups (ACT versus mEUC) and four repeated measures of variance (ANOVA) with within-between interaction (group x time) using the G*Power calculator [59] and simulation study of the time course with dichotomous between-person level predictor [60]. Given the large effects of ACT on psychological well-being, including hope (Hedge’s *g* = 0.88–2.17) and medium effects on psychological flexibility among cancer patients (Hedge’s *g* = 0.58) [29], the stronger effects in the population of women with breast cancer compared to patients with other types of cancer (large versus medium effect sizes) [31], and medium effect sizes of technology-supported ACT interventions (Hedges’ *g* = 0.44–0.48) [21], moderate differences between conditions were expected. Assuming a medium effect size of *f* = 0.25, a power of 0.80, and an alpha level of 0.05 in repeated measures of ANOVA, a total sample size of *N* = 178 is required. In turn, on the basis of a simulation study, a total sample size of *N* = 136 is required for multilevel modeling. Therefore, the minimum sample size was assumed of *N* = 160 (80 per condition). Allowing for the potential attrition rate of 20%, this leads to a sample size of *N* = 192 participants, including 96 in each arm. In SCED, 6–9 participants will be investigated, a minimum of 2 per condition. According to the simulation study [61], sufficient power (0.80) can be reached in SCED with six to eight participants, depending on the assumed effect size (large versus medium, respectively).

### Recruitment {15}

**Fig. 2:**
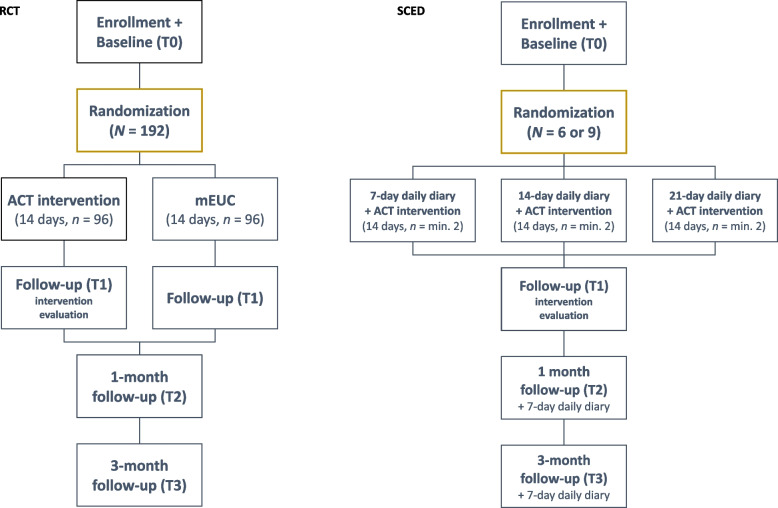
Participant flowchart in RCT and SCED study. ACT, Acceptance and Commitment Therapy; mEUC, minimally enhanced usual care

Recruitment will take place at a single center, after elective admission to the bone marrow transplantation and oncohematology unit due to HCT before the start of conditioning treatment. Recruitment will take place on average on the 2nd day after admission. Every 2 days, the transplant coordinator, PI, and physician (members of the research team) will review the lists of patients enrolled for HCT. Those who meet the inclusion criteria will be initially informed of the purpose of the study and invited for an extensive briefing by a recruiter (member of the research team). Patients will also be allowed to ask any remaining questions about the aim of the study and the study procedures. After receiving an extensive briefing, all patients who give written informed consent will proceed with baseline. Recruitment will be carried out until the desired sample size is achieved. The flowchart of the study is depicted in Fig. 2.

## Assignment of interventions: allocation

### Sequence generation {16a}

The allocation sequence will be generated using the method of minimization. Minimization can be classified as dynamic allocation or covariate adaptive methods because the allocation depends on the characteristics of the patients and is performed continuously [62]. Randomization will be stratified by type of transplant (autologous versus allogeneic) to ensure a balanced representation between the study conditions because autologous and allogeneic HCT recipients experience different recovery trajectories and HCT impact on well-being [63, 64].

### Concealment mechanism {16b}

The mechanism of implementing the allocation sequence will be central randomization. It means generating an allocation sequence after the patient is enrolled [65]. This way, randomization will not affect the recruitment process.

### Implementation {16c}

The trial coordinator (member of the research team) will enroll participants, generate the allocation sequence, and assign participants to interventions. Other members of the team will be blind to the allocation of the participants to the conditions.

## Assignment of interventions: blinding

### Who will be blinded {17a}

In RCT, trial participants, care providers, outcome assessors, and data analysts will be blinded after assignment to interventions. Blinding will be performed using two separate databases: one containing participant allocation information (blinded) and the other containing the remaining information (unblinded). Only the trial coordinator will have access to the blinded database.

### Procedure for unblinding if needed {17b}

Disclosure of the participant allocation will take place after the completion of the study and analysis of the first results examining the efficacy of the online ACT intervention.

## Data collection and management

### Plans for assessment and collection of outcomes {18a}

Data will be collected via self-reported online questionnaires at the baseline (before HCT), post-intervention, and 1 and 3-month follow-ups (Table 2). In addition, to assess momentary changes and mechanisms of change, participants will complete daily diaries throughout the intervention. SCED participants will complete 7-day daily diaries repeatedly, i.e., before 1 and 3-month follow-ups. The detailed characteristics of the study instruments are presented in Table 3.

We intend to collect clinical data (e.g., diagnosis, time from diagnosis, type of transplant and conditioning treatment, comorbidities) from the patient’s medical records. The participants will give their additional consent for the data to be collected from their medical history by a physician (team member). If the participant does not approve of access to the data from medical records, they will be requested to provide information themselves.

### Plans to promote participant retention and complete follow-up {18b}

To improve participant retention and complete follow-up, participants will receive email and phone reminders about the survey and subsequent measurements. If participants fail to complete study assessments, motivational reminders will be sent repeatedly by email. In daily diary measurements, participants who give written consent will receive SMS reminders. Since the daily diaries will not be filled retrospectively, a single reminder with the mailing of the survey will be used.

During the study, direct technical support will be available 24/7, and a research team member will contact the participant by phone to resolve any issues and answer questions. If participants drop out of the study, they will be asked for the reason(s). Any other attritions (e.g., disqualification from HCT, death) along with the reasons will be recorded.

### Data management {19}

Questionnaire data collection will be done electronically (using the SurveyMonkey platform, which encrypts and secures data during transit and the data stored; the accounts are password-protected with available complexity controls). Medical data will be collected electronically directly from the medical records registry by the physician (member of the research team). Only informed consents will be paper documents, collected and entered by recruiter (member of the research team). The PI will be responsible for the secure delivery of the documents to the trial office. The PI and trial coordinator will oversee the quality of the data. Data and metadata storage will take place in the university’s central resources according to the 3–2-1 rule. The detailed data management plan is available at OSF.

### Confidentiality {27}

Personal data such as phone numbers and email addresses of the participants will be encrypted (using individual trial identification number) and stored only during the data collection period. Written informed consent and the data identifying the participants will be stored separately under lock and key and will be kept strictly confidential. The data will be accessed by the PI of the project and selected team members who will be contacting the participants (trained in the General Data Protection Regulation). Access to the data will be monitored and possible only after obtaining the access rights that the PI of the project will grant. Once data collection is completed, the data will be anonymized and in this form will be analyzed statistically.

### Plans for collection, laboratory evaluation and storage of biological specimens for genetic or molecular analysis in this trial/future use {33}

N/a. Biological specimens will not be collected.

## Statistical methods

### Statistical methods for primary and secondary outcomes {20a}

Analyses will be conducted using the latest Mplus statistical package [66], R [67], and IBM SPSS (IBM Corp.; Armonk, NY). We will use the standard *α* = 0.05 or 95% confidence interval for the determination of value probability. All data analysis will be performed according to the intention-to-treat principle, where all randomized participants are included in the analysis assuming missing data at random. The collected data will be first analyzed in terms of sample characteristics and comparisons (frequency, descriptive statistics; ANOVA, *t*-test or their nonparametric counterparts; *χ*^2^; Pearson’s or Kendall’s correlation), missing data (frequency, multilevel modeling), and sample attrition (logistic regression analysis). Multilevel confirmatory factor analysis (MCFA) will be performed to establish the respective measurement models and calculate the indicator reliabilities (omega coefficient) at the within- and between-person levels [60, 68]. To examine hypotheses 1–3, latent curve growth modeling (LCGM) [69] and multilevel (MSEM) and dynamic structural equation modeling (DSEM) will be applied [60, 70]. All methods allow for the examination of the time course. In addition, MSEM and DSEM allow for the calculation of simple between- and within-person associations and more advanced associations such as mediations and moderations. Hypotheses 4–7 will be verified using a multilevel vector autoregressive (mlVAR) model [71]. mlVAR allows for the examination of a temporal network (i.e., lagged predictive relations between each node in the network and each node in the network at the next measurement occasion), a contemporaneous network (i.e., partial correlations within the same measurement occasion), and a between-person network (i.e., associations between nodes that are averaged across measurement occasion).

### Interim analyses {21b}

Due to a known minimal risk, i.e., testing interventions with known positive effects, an interim analysis plan was not created. The principal investigator (PI) will make the final decision to terminate the study once the optimal number of study participants has been obtained.

### Methods for additional analyses (e.g., subgroup analyses) {20b}

All analyses will be supplemented by sensitivity analyses. In all models, possible confounders (i.e., demographics, clinical factors, and other confounders) will be considered after preliminary selection.

### Methods in analysis to handle protocol non-adherence and any statistical methods to handle missing data {20c}

The statistical methods used (i.e., MSEM, DSEM) will allow the most recent flexible approach to the missing data (the full information maximum likelihood) [72, 73]. In less sophisticated analyses, missing data will be multiple imputed in advance.

### Plans to give access to the full protocol, participant-level data and statistical code {31c}

The full protocol, dataset, statistical codes, and outputs will be made available at the Open Science Framework (OSF). Participant-level datasets will be publicly available, however without demographics and clinical data due to privacy or ethical restrictions (the possibility of identification of participants).

## Oversight and monitoring

### Composition of the coordinating center and trial steering committee {5d}

The study’s coordinating center is SWPS University. The study’s steering committee will consist of a health psychologist, a certified cognitive behavioral therapist (CBT) and ACT therapist, and a doctoral student (master’s degree in psychology). The committee’s responsibilities will be to develop the intervention and then implement it and monitor implementation. The committee will meet 2–4 times a month.

### Composition of the data monitoring committee, its role and reporting structure {21a}

Due to known minimal risks, a formal committee of data monitoring is not needed.

### Adverse event reporting and harms {22}

In this study, an adverse event will be defined as any deterioration in mood that requires specialized treatment, collected after the individual has received the intervention, and reported to the local institutional review board (IRB).

### Frequency and plans for auditing trial conduct {23}

No audit procedures are planned. An independent audit may be conducted by the local IRB and the sponsor.

### Plans for communicating important protocol amendments to relevant parties (e.g., trial participants, ethical committees) {25}

Communication of significant protocol modifications and study outcomes will be done to the funder, the ethics committee, and the public through ClinicalTrials.gov.

### Dissemination plans {31a}

The results will be published in peer-reviewed journals and presented at thematic international scientific conferences. Also, during the debriefing, participants will be informed of the web address of the project website, where a lay summary of the study updated with the results (when available) will be posted.

## Discussion

Effective treatment of patients undergoing HCT likely requires a focus also on those mechanisms that support the reconstruction of meaning damaged by medical treatment and the disease itself. An intervention based on ACT, an empirically validated theoretical model [17], appears to be a promising psychological therapy to support the reconstruction of meanings [33, 34]. This trial represents the first study that aims to integrate the ACT and meaning-making frameworks to reduce meaning-related distress, stimulate the meaning-making process, and enhance the well-being of HCT recipients. It builds on previous successful ACT interventions that strengthened cancer patient well-being albeit outside the context of meaning reconstruction [25–29]. Moreover, testing a specific theory-based intervention to address existential concerns unique to patients undergoing HCT will be reinforced by a statistically rigorous idiographic approach. SCED will allow us to go beyond aggregate group effects and see how a specific person responds to an ACT intervention, thereby providing clinical input into *what* works for *whom* and *when*. Beyond this, since access to interventions in the HCT population is limited, the web-based ACT self-help program we designed has the potential to fill that gap. Self-directed ACT interventions are considered cost-effective, flexible, and accessible for cancer patients [21]. They allow patients to self-determine what (content), when (time), where (location), and how (read or listen) to use ACT intervention booklets.

Despite these strengths, we expect several challenges and limitations. First, recruiting the HCT recipients will be challenging. Therefore, we allow for the possibility of recruiting at a second oncohematology center with identical credentials. Retaining participants in the study can be also a challenge, hence the contact maintenance and participation reminder activities we have planned. In addition, we plan to compensate participants for their participation at a rate of PLN 150 (approx. 34.5 Euros) in RCT and PLN 300 (approx. 69 Euros) in SCED. Another limitation is the targeting of the trial to all willing HCT recipients, regardless of the level of distress or the stage of the meaning reconstruction process. However, we are guided by pragmatic (restrictive inclusion/exclusion criteria would prolong the already long data collection time) and cognitive considerations (to our knowledge, this is the first study that will test the relationship of ACT interventions to meaning reconstruction processes) hoping that this will result in further research in this area.

## Trial status

ClinicalTrials.gov, NCT06266182. Registered 20 February 2024, https://clinicaltrials.gov/study/NCT06266182. Version 3.0 dated May 13, 2024. Patient recruitment began on March 6, 2024. Recruitment is expected to be completed in December 2025.

## Data Availability

Data that will be collected during the current study (without demographics and clinical data due to the possibility of identification of participants), full protocol, statistical codes, and outputs will be made available at the Open Science Framework (OSF).

## Abbreviations

ACT: Acceptance and Commitment Therapy
ANOVA: Analysis of variance
B-IPQ: Brief-Illness Perception Questionnaire
CBI: Core Beliefs Inventory
CBT: Cognitive behavioral therapy
CompACT-9: Comprehensive Assessment of Acceptance and Commitment Therapy Processes
C-PTGI-SF: The “current standing” Post-Traumatic Growth Inventory-Short Form
CSE: Coping Self-Efficacy Scale
CSQ-8: Client Satisfaction Questionnaire
DiMIS: Diversity Minimal Item Set
DSEM: Dynamic structural equation modeling
EORTC QLQ-C30: Quality of Life Questionnaire of the European Organization for Research and Treatment of Cancer
ERRI: Event-Related Rumination Inventory
GMVS: Global Meaning Violation Scale
HCT: Hematopoietic cell transplantation
IRB: Institutional review board
LCGM: Latent curve growth modeling
MCFA: Multilevel confirmatory factor analysis
mEUC: Minimally enhanced usual care
MLC: Meaning of Loss Codebook
MLQ: Meaning in Life Questionnaire
mlVAR: Multilevel vector autoregressive
MSCNRIO: Maria Sklodowska-Curie National Research Institute of Oncology
MSEM: Multilevel structural equation modeling
OSF: Open Science Framework
PHQ-4: Patient Health Questionnaire
PI: Principal investigator
RCT: Randomized controlled trial
R-UCLA: Revised UCLA Loneliness Scale
SCED: Single-case experimental design
